# Energy metabolism of pregnant zebu and crossbred zebu dairy cattle

**DOI:** 10.1371/journal.pone.0246208

**Published:** 2021-02-04

**Authors:** Helena Ferreira Lage, Ana Luiza da Costa Cruz Borges, Ricardo Reis e Silva, Alan Maia Borges, José Reinaldo Mendes Ruas, Pedro Henrique Araújo de Carvalho, Marcelina Pereira da Fonseca, Paolo Antônio Dutra Vivenza, Lúcio Carlos Gonçalves, André Santos de Souza, Antônio Último de Carvalho, Elias Jorge Facury Filho, Edilane Aparecida Silva, Joana Ribeiro da Glória, Alexandre Lima Ferreira, Rodrigo Melo Meneses, Eloísa de Oliveira Simões Saliba

**Affiliations:** 1 Veterinary School, Universidade Federal de Minas Gerais, Belo Horizonte, Minas Gerais, Brazil; 2 Departament of Animal Science, Unimontes, Janaúba, Minas Gerais, Brazil; 3 Empresa de Pesquisa Agropecuária de Minas Gerais, Uberaba, Minas Gerais, Brazil; University of Illinois, UNITED STATES

## Abstract

The purpose of this study was to determine the energy partition of pregnant F1 Holstein x Gyr with average initial body weight (BW) of 515.6 kg and Gyr cows with average initial BW of 435.1 kg at 180, 210 and 240 days of gestation, obtained using respirometry. Twelve animals in two groups (six per genetic group) received a restricted diet equivalent to 1.3 times the net energy for maintenance (NE_m_). The proportion of gross energy intake (GEI) lost as feces did not differ between the evaluated breeds and corresponded to 28.65% on average. The daily methane production (L/d) was greater for (P<0.05) F1 HxG compared to Gyr animals. However, when expressed as L/kg dry matter (DM) or as percentage of GEI there were no differences between the groups (P>0.05). The daily loss of energy as urine (mean of 1.42 Mcal/d) did not differ (P>0.05) between groups and ranged from 3.87 to 5.35% of the GEI. The metabolizable energy intake (MEI) of F1 HxG animals was greater (P < 0.05) at all gestational stages compared to Gyr cows when expressed in Mcal/d. However, when expressed in kcal/kg of metabolic BW (BW^0,75^), the F1 HxG cows had MEI 11% greater (P<0.05) at 240 days of gestation and averaged 194.39 kcal/kg of BW^0,75^. Gyr cows showed no change in MEI over time (P>0.05), with a mean of 146.66 kcal/kg BW^0. 75^. The ME used by the conceptus was calculated by deducting the metabolizable energy for maintenance (ME_m_) from the MEI, which was obtained in a previous study using the same cows prior to becoming pregnant. The values of NEm obtained in the previous study with similar non-pregnant cows were 92.02 kcal/kg BW^0.75^ for F1 HxG, and 76.83 kcal/kg BW^0.75^ for Gyr (P = 0.06). The average ME for pregnancy (ME_p_) was 5.33 Mcal/d for F1 HxG and 4.46 Mcal/d for Gyr. The metabolizability ratio, averaging 0.60, was similar among the evaluated groups (P>0.05). The ME / Digestible Energy (DE) ratio differed between groups and periods evaluated (P<0.05) with a mean of 0.84. The heat increment (HI) accounted for 22.74% and 24.38% of the GEI for F1 HxG and Gyr cows, respectively. The proportion of GEI used in the basal metabolism by pregnant cows in this study represented 29.69%. However, there were no differences between the breeds and the evaluation periods and corresponded to 29.69%. The mean NE for pregnancy (NE_p_) was 2.76 Mcal/d and did not differ between groups and gestational stages (P>0.05).

## Introduction

There is no doubt that within the production process of livestock, females are the most challenged individuals because of the constant physiological changes to which they are exposed. Among these changes, lactation has been extensively studied in ruminants in recent years [1, 2] because of its importance to ensure the health of the progeny, as well as its economic importance in providing raw material for the dairy industry. However, pregnancy, which is the "pre-requisite" for lactation to occur, has been somewhat overlooked in animal science studies, especially for understanding pregnant cow’s metabolism. [3] reported data of energy metabolism and nutritional requirements during pregnancy of cows and considered the understanding of gestation as a major aspect for ensuring the mother’s health and proper development of the fetus. On the other hand, the same authors mentioned the limitation of scientific information available on this topic and 40 years later, the statements of these authors can still legitimately be considered, especially regarding the information on energy metabolism of pregnant zebu cows and their crosses. In the 90s, [4] added valuable information about the development of pregnancy by evaluating the energy content of the uterus at various gestational stages. The authors stipulated that until 190 days gestational age, an increase in the energy requirement would be insignificant. Therefore, it should not be considered in the formulation of diets for dairy cows [5]. Recently, a study presented data for energy requirements of crossbred F1 Holstein x Gyr cows [6]. Although the study of [6] represents a major advance in the study of energy metabolism of pregnant cows used in tropical conditions, the slaughter methodology adopted has some limitation regarding energy efficiency and detail the energy partition by animals. In this work, using respirometry technology, we intend to describe the energy partition, energy requirements and efficiency of energy utilization by pregnant F1 Holstein x Gyr (F1 HxG) and Gyr cows at 180, 210 and 240 days of gestation.

## Materials and methods

The experimental protocol followed the guidelines for the use of animals for scientific purposes in Brazil and was approved by the Ethics and Animal Experimentation Committee of the Universidade Federal de Minas Gerais (protocol number 184–2015).

The experiment was carried out in the Animal Metabolism and Calorimetry Laboratory (LAMACA) of the Department of Animal Sciences at the School of Veterinary Medicine, Universidade Federal de Minas Gerais, Belo Horizonte, MG, Brazil. The present study involved twelve primiparous pregnant cows, six F1 Holstein x Gyr (F1 HxG) and six Gyr, all inseminated with the same Holstein bull semen. All animals were from the Experimental Farm of the Minas Gerais Agricultural Research Company (EPAMIG), located at Felixlandia (MG, Brazil). The initial body weight (BW) averages were 515.6 ± 81 kg and 435.2 ± 49 kg, respectively for crossbred and zebu females. The experimental cows were prepared for use in energy balance studies. During the experiment, the animals were confined in tie-stall type installations containing cover and rubber pallets on the floor to ensure comfort for the animals. The facilities allowed the daily exposure of animals to sunlight and free access to individual water. The experimental diets are shown in Table 1.

**Table 1:** Composition of the experimental diets provided for F1 HxG an Gyr cows at 180, 210 and 240 days of gestation.

Item	Gestational age (days)		
	180	210	240
**Ingredient, % DM**			
Corn silage	93.3	82.5	81.75
Soybean meal	6,20	17.10	17,75
Comercial Mineral premix	0.5	0.5	0.5
**Chemical composition**			
OM, % DM	91.02	91.86	91.01
CP, % DM	10.51	15.42	15.24
EE, % DM	2.16	2.22	2.40
NDF_ap_^1^, % DM	55.00	50.00	49.70
NFC^2^, % DM	21.04	21.94	22.16
TDN^3^, % DM	60.20	67.30	65.81
Ca, % DM	0.65	0.67	0.64
P, % DM	0.39	0.46	0.43
ME^4^, Mcal/kg	2.68	2.76	2.74
NE^5^, Mcal/kg	1.62	1.63	1.78

Means followed by different lowercase letters in the same row differ between times evaluated in the same genetic group. Means followed by different capital letters in the same row differ between genetic groups evaluated at the same gestational age.

^1^NDF_ap_ = Neutral detergent fiber corrected for ash and protein.

^2^NFC = non-fiber carbohydrate.

^3^NDT = determined by [7].

^4^ME = average metabolizable energy obtained by energy partition.

^5^NE = average net energy obtained by the energy partition.

There was a period of initial adaptation to the experimental diet, from day 120 to day 180 of gestation. The objective of the adaptation period was to ensure that all animals consumed sufficient food to maintain BCS of 3.5 and controlled average daily gain (ADG) to ensure minimal maternal energy retention (maternal tissue deposition). There were individual adjustments to the dry matter intake (DMI) based on the ADG observation by weighing and body condition score evaluation (BCS) [8] of each animal, once every two weeks. We avoided creating energy restrictions of the dams that could cause tissue mobilization or impair foetal development. From the 180th day of pregnancy, the acquisition of *in vivo* data was initiated with experimental diets (Table 1) that were formulated to provide energy of 1.2 times the net energy for maintenance (NE_m_).

According to [4], the [5] postulates that the demand for energy and nutrients for pregnancy only becomes significant from the 190th day. The experiment was scheduled so that the evaluation of the animals could be made at 180, 210 and 240 days of gestation. Since the conception of each cow occurred on different dates, a tiered system was arranged so that the *in vivo* data was acquired at 180 ± 6, 210 ± 7 and 240 ± 6 days of gestation.

### Digestibility trial and urine collection

Daily feed consumption and faecal daily production was measured by collecting the feed offered and total faeces for five consecutive days. All feed and faeces were collected, weighed, and sampled daily. Due to the restricted amount of food provided, to ensure adequate weight gain associated with the gravid uterus, there were no orts to be sampled. Samples of 400 grams were placed in plastic bags and frozen at -15°C for later analysis.

After the digestibility trials and calorimetry measurements, a single urine collection was performed at 250^th^ day of gestation in order minimize the risk of bladder injurie or infection. For the total collection of urine, a urethral catheter (Foley, n° 26) was inserted and fixed in the urethra. Once inserted and fixed, the catheter was connected to silicone hose of approximately two meters length. The free end was attached to a sanitized container containing 500ml of 20% sulfuric acid so that the pH of the final urine plus acid solution was below 2.0, avoiding urinary nitrogen volatilization. Each animal remained catheterized for 72h (24h for adaptation and 48h for total urine collection) to measure the total urine volume. Daily urine output was considered as the sum of the 48h urinary production and the value obtained for each animal was used to estimate the amount of energy lost as urine at 180^th^, 210^th^ and 240^th^ day of gestation.

### Calorimetry

Right after each apparent digestibility trial, methane and heat production were measured for 22 to 23 hours by performing extrapolation for a period of 24 h, using an open-circuit indirect calorimetry system, adopted by the Veterinary School, UFMG, according to [9, 10]. The respirometric chamber was made of steel with acrylic side windows, 3.45 m long, 1.45 m wide and 2.45 m high (22.391L of internal volume). The chamber has a sealing system which prevents any gas leaks from the inside chamber. In this system, the air inside the chamber is continuously renewed by air from outside the chamber, by an external pump with a specific renovation rate (1.0 litre gas/kg BW/minute) to avoid concentration of CO_2_ more than 1.0%. Samples of atmospheric air (baseline gas) and of air inside the respirometric chamber are repeatedly collected every 5 minutes by a specific pumping system and redirected to the Sable Systems (Las Vegas, NV, USA) gas analysers. Then, each gas sample is analysed for concentrations of oxygen, carbon dioxide, and methane. The system requires 1 to 2 hours of daily calibration of gas analysers. The chamber was opened every 24h to clean and to collect any residues of feed. The afternoon feed (16:00h) was done through an automatic feeding window. This procedure was performed for about 1 minute during an interval when no samples of air inside the chamber was being collected. Otherwise, the feeding process could interfere with the concentrations of the gases inside the chamber. The respirometric chamber was kept between 22 and 26°C and between 65 and 80% relative humidity. Each animal was individually analysed and their daily heat production (HP) of the was determined using the equation proposed by [11]:

$$\text{HP}(\text{kcal})=(16.18\;{\text{O}}_{2}+5.02\;{\text{CO}}_{2}-2.17\;{\text{CH}}_{4}-5.99\;\text{N})\;/\;4.184$$

Where volume of O_2_ consumed (L/d), volume of CO_2_ produced (L/d), volume of CH_4_ produced (L/d) and N the amount of nitrogen excreted in the urine (g/d) are considered.

### Chemical analysis

Samples of feed and faeces were defrosted at room temperature and pre-dried at 55 ± 5°C for 72 hours [12]. Initially, they were ground using a stationary type Thomas-Willey mill with a five mm mesh sieve. The 5-mm milled samples were than proportionally mixed to produce only one sample that represented each animal at each digestibility trial. Subsequently, each mixed sample was ground one more time in a stationary mill with a 1-mm mesh sieve and stored in a polyethylene flask for chemical analysis. The content of dry matter (DM) was determined at 105°C (proc. 930.15 [13]). The content of organic matter (OM) was calculated as the difference between the DM contents and ash content, with ash content determined by combustion at 600°C for 4 hours. The crude protein content (CP, 6:25 x nitrogen) was measured using the Kjeldahl method (proc. 976.05; [13]); the ether extract (EE) was measured using to the Soxhlet method (proc. 963.15; [13]). The neutral detergent fibre corrected for ash and protein content (NDFap) was determined using a Fiber Analyzer ANKOM^®^ device (Ankom^™^ Technology, Fairport, NY, USA), using the serial method, described by [14]. The non-fibrous carbohydrates (NFC) were calculated using the equation proposed by [5] as follows:

NFC=100−(%CP+%NDFap+%EE+%ash).

The total nitrogen of the *in natura* urine samples was determined by the Kjeldahl method (proc. 976.05; [13]). The GE content of the feed, faeces and urine samples was determined by combustion using a PARR 2081 adiabatic bomb calorimeter.

### Energy balance calculations

The daily gross energy intake (GEI) was determined by multiplying the caloric content of the feed by the total feed intake. The DE intake (DEI) was calculated from the difference between the GEI and the amount of energy lost as feces (E_feces_).

$$\text{DEI}(\text{Mcal}/\text{d})=\text{GEI}-{\text{E}}_{\text{feces}}$$

The ME intake (MEI) was obtained by subtracting the energy lost as urine (E_urine_) and methane production from the DEI (E_CH4_).

$$\text{MEI}=\text{DEI}–\text{E}\;\text{urine}–{\text{E}}_{\text{CH}4}$$

The urinary energy content was determined by the total combustion of samples using an adiabatic calorimeter, and this value was applied to the average daily urine production. Energy lost as methane was quantified by multiplying the daily production of methane by the caloric equivalent of 9.45 kcal/L of methane [11]

To obtain the net energy (NE) values, it was necessary to measure the energy lost as heat from basal metabolism (fasting heat production, FHP) and the heat from the heat increment of the diet (HI). The heat from fasted animals was previously determined during a period of 72h of fasting, which corresponds to the net energy for maintenance (NE_m_) of the same cows prior to impregnation [15]. The NE_m_ were, respectively, 92.02 and 76.83 kcal/kg of metabolic BW (BW^0,75^) for F1 HxG and Gyr animals. The efficiencies of 0.63 and 0.64 were used for F1 HxG and Gir cows, respectively [15], to determine the ME for maintenance (ME_m_). With those values, we found 120.05 kcal/kg BW^0.75^ for Gyr cows and 146.06 kcal/kg BW^0.75^ for the crossbred cows.

To spare the pregnant F1 HxG and Gyr cows from stress promoted by fasting, the HI associated with digestive functions was determined using six adult, non-pregnant, nulliparous Zebu dairy cows with an average BW of 594 kg. These animals received the same experimental diets and the DMI level, set at 1.5% of BW, was equal to that used for pregnant F1 HxG and Gyr cows. Those cows also had their FHP measured using the respirometry system described above. The HI of the diet was obtained from the difference between the HP of fed animals and the FHP.

$${\text{HI}}_{\text{diet}}=\text{Fed}\;\text{HP}-\text{FHP}$$

In pregnant females, heat production by gravid uterus should also be considered as a source of energy loss. This variable was obtained from difference between the pregnant fed HP from the FHP and HI.

$${\text{HP}}_{\text{gravid}\;\text{uterus}}={\text{HP}}_{\text{fed}}–\text{FHP}–\text{HI}$$

The metabolizability (q) of the diet was calculated by divining MEI by GEI as proposed by the [16]:

q=MEI/GEI

The efficiency of utilization of ME for pregnancy (k_p_) corresponded to the ratio between NE for pregnancy (NE_p_) and ME for pregnancy (ME_p_).

$$\text{kp}={\text{NE}}_{\text{p}}/{\text{ME}}_{\text{p}}$$

### Statistical analysis

The statistical design was *split-plot* arrangement, in which breeds represent parcels and gestational ages correspond to the sub-plots. The variables were subjected to variance analysis (ANOVA) using the SAS software [17] assuming 5.0% as the critical level of probability. Interaction between genetic group and gestational ages as the differences between treatments were considered significant at P < 0.05. Tukey test was used for comparisons between means.

## Results and discussion

The DMI was greater for F1 H x G cows in all the evaluated gestational ages, possibly because of their higher BW, as can be seen in Table 2. However, when measured as a percentage of BW there were no difference in the DMI between the genetic groups. Even when expressed as a function of metabolic body weight (BW^0,75^), DMI was greater for F1 HxG cows only at 240 days of gestation.

**Table 2:** Body weight (BW) and dry matter intake (DMI) of F1 Holstein x Gyr (F1 HxG) and Gyr cows at 180, 210 and 240 days of gestation.

Item	Genetic group / Gestational age (days)						GG*GA^3^
	F1 HxG			Gyr			
	180	210	240	180	210	240	
BW, kg	515.60^a^	538.40^b^	568.40^Bc^	435.16^a^	455.83^b^	469.33^Ab^	NS^4^
DMI, kg/d	7.82^Ab^	7.73^Ab^	8.82^Aa^	6.11^B^	5.71^B^	6.11^B^	NS^4^
SEM^2^	0.17	0.53	0.31	0.43	0.52	0.33	
DMI, % BW	1.55	1.46	1.56	1.43	1.26	1.30	NS^4^
SEM^2^	0.12	0.12	0.07	0.14	0.12	0.08	
DMI, g/kg BW^0.75^ ^(1)^	73.52	69.94	76.24^A^	64.97	58.22	60.83^B^	NS^4^
SEM^2^	4.62	5.31	2.96	5.71	5.24	3.24	

Means followed by different lowercase letters in the same row differ between times evaluated in the same genetic group. Means followed by different capital letters in the same row differ between genetic groups evaluated at the same gestational age.

^1^BW^0.75^ = metabolic body weight (BW^0,75^).

^2^SEM = standard error of mean.

^3^GG*GA = interaction between genetic group and gestational age.

NS = non-significative (P>0.05).

The variations in the daily DMI were controlled according to periodic weighing and BCS of the animals. Just subtle adjustments in consumption were required, because the BCS of cows at the start of data acquisition (at 180 days of gestation) was already standardized and considered adequate (BCS = 3.5). In the present study those considerations between level of intake and nutritional requirements are critical because the NE_m_ was determined previously by [15] with the same non-pregnant F1 HxG and Gyr cows consuming restricted amount of fed. Several studies have also shown an association between feeding level and size of viscera in pregnant cows [18–21]. Although the visceral organs correspond to a low proportion of animal BW (11 to 12%, with inclusion of visceral fat and 5 to 6% without), it is important to evaluate the influence of DMI on the size and activity of viscera because they require about 40 to 50% of the NE_m_ [22]. The level of intake was set to ensure no modifications on NE_m_ determined by [15]. In addition, during the experiment with pregnant animals, it was not possible to isolate the maternal gain from the gain of the development of conceptus. A high feeding level could lead to high maternal tissue deposition which was undesirable for the determination of energy requirements for pregnancy in this experiment. ADG during the total experimental period was similar (P>0.05) between crossbred and Zebu cows being 0.507 kg/d for F1 HxG cows and 0.472 kg/d for Gyr cows. The equation proposed by [4] determine that the wet weight gain of the gravid uterus between 190 and 240 day of gestation should be 0.66 kg/d for Holstein multiparous cows (mean BW 714 ± 16 kg) with a calf birth weight projected to be 46 kg. Although the equation proposed by these authors was developed for dairy *Bos taurus* cattle, it was used to better understand the magnitude of the maternal weight gain since there is a limitation in the literature regarding this topic. The slightly lower BW gain presented in the animals seems to be adequate, since the birth weight is less for crossbred and zebu cattle. The mean birth weight for crossbred F1 Hx G calf of 35 kg was obtained by [23].

Evaluating the effects of energy restriction and protein on Holstein cows during the last three weeks of pregnancy, [24] reported that a consumption of 1.50% of BW would be enough to meet the mother´s maintenance requirements and proper foetal development with no positive or negative energy balance. The DMI obtained for the F1H x G did not differ (P>0.05) among the gestational ages, on average, was equivalent to 1.52% of the live weight of the animals. The Gyr cows also showed similar DMI between gestational ages with DMI numerically lower (P>0.05) than crossbred cows with an average of 1.33% of BW. The data presented in DMI shows some evidence that, if it is intended to maintain the same body condition, it was necessary that the zebu cows consume less food than the crossbred cows, probably due to their lower (P = 0.06) NE_m_ (92.02 and 76.83 and kcal/kg BW^0.75^ for F1 HxG and Gyr, respectively) as described by [15].

As with the DMI, the GEI (Mcal/d) of F1 HxG cows was greater (P<0.05). The daily energy loss as feces (Mcal/d) was also greater (P<0.05) in crossbred cows compared to Gyr animals at all gestational ages (Table 3). However, the percentage of GEI lost as fecal output did not differ over time or among genetic groups.

**Table 3:** Energy partition of F1 Holstein x Gyr (F1 HxG) and Gyr cows at 180, 210 and 240 days of gestation.

Item	Genetic group (GG) / Gestational age, days (GA)						GG*GA^13^
	F1 HxG			Gyr			
	180	210	240	180	210	240	
GE^1^, Mcal/d	34.30^Ab^	35.10^Ab^	39.84^Aa^	27.00^B^	25.84^B^	26.96^B^	NS^14^
SEM^2^	1.01	2.17	1.42	1.36	2.08	1.37	
Feces, Mcal/d	10.82^A^	9.76^A^	11.12^A^	7.78^B^	6.91^B^	8.09^B^	NS^14^
SEM^2^	1.24	1.14	0.38	0.56	0.72	0.47	
DE^3^, Mcal/d	23.48^Ab^	25.34^Ab^	28.72^Aa^	19.21^B^	18.92^B^	18.86^B^	NS^14^
SEM^2^	0.49	1.05	1.21	0.87	1.53	0.98	
Methane (CH_4_), Mcal/d	2.01^Ab^	2.60^Aa^	2.53^Aa^	1.44^Bb^	1.73^Bab^	1.86^Bb^	NS^14^
SEM^2^	0.07	0.21	0.16	0.07	0.16	0.19	
Urine, Mcal/d	1.78	1.78	1.78	1.06	1.06	1.06	NS^14^
ME^4^, Mcal/d	19.68^Ab^	20.95^Ab^	24.40^Aa^	16.70^B^	16.13^B^	15.93^B^	NS^14^
SEM^2^	0.46	0.88	1.19	0.75	1.25	0.78	
ME_m_^5^, Mcal/dia	15.76^Ac^	16.28^Ab^	16.97^Aa^	11.42^Bb^	11.83^Ba^	12.09^Ba^	NS^14^
SEM^2^	0.76	0.83	0.30	0.40	0.39	0.68	
ME_p_^6^, Mcal/d	3.92^b^	4.66^b^	7.42^Aa^	5.27	4.3	3.83^B^	NS^14^
SEM^2^	0.42	0.91	0.97	0.98	1.29	0.79	
Fed HP^7^, Mcal/d	18.51^b^	20.72^Aa^	22.91^Aa^	16.10	16.00^B^	15.64^B^	< 0.05
SEM^2^	0.46	0.51	0.41	0.25	0.24	0.18	
FHP^8^, Mcal/d	9.55^Ab^	9.87^Ac^	10.29^Bb^	7.03^Bb^	7.28^Ba^	7.45^Ba^	NS^14^
SEM^2^	0.46	0.51	0.41	0.25	0.24	0.18	
HI^9^, Mcal/d	7.73	8.89^A^	8.08^A^	6.65	6.75^B^	5.97^B^	NS^14^
SEM^2^	0.28	0.31	0.99	0.56	0.50	0.65	
Total NE^10^, Mcal/d	11.94^b^	12.05^Ab^	16.32^Aa^	10.04	9.37^B^	9.95^B^	NS^14^
SEM^2^	0.27	0.71	0.53	0.61	1.00	0.62	
HP_p_^11^, Mcal/d	1.21^b^	1.95^b^	4.54^Aa^	2.40	1.96	2.21^B^	NS^14^
SEM^2^	0.11	0.59	0.62	0.54	0.90	0.54	
NE_p_^12^, Mcal/d	2.70	2.71	2.88	2.86	2.33	1.62	<0.05
SEM^2^	0.48	0.40	1.35	0.60	0.59	0.70	

Means followed by different lowercase letters in the same row differ between times evaluated in the same genetic group. Means followed by different capital letters in the same row differ between genetic groups evaluated at the same gestational age.

^1^GE = gross energy.

^2^SEM = standard error of mean.

^3^DE = digestible energy.

^4^ME = metabolizable energy.

^5^ME_m_ = metabolizable energy for maintenance [15].

^6^ME_p_ = metabolizable energy for pregnancy (ME_p_ = MEI–ME_m_).

^7^Fed HP = fed heat production determined in open-circuit calorimetry system.

^8^FHP = fasting heat production determined by open-circuit calorimetry system [15].

^9^HI = heat increment obtained by the difference between Fed HP and FHP determined in non-pregnant Zebu dairy cows consuming the same experimental diets.

^10^Total NE = total net energy intake.

^11^HP_p_ = heat production of the conceptus (HP_p_ = Fed HP–FHP–HI).

^12^NE_p_ = net energy for pregnancy.

^13^GG*GA = interaction between genetic group and gestational age.

^14^NS = non-significative (P>0.05).

Hereford heifers described by [3], consuming equivalent to 1.3 x NE_m_ requirements, had fecal losses equivalent to 30.01% and 26.65% of GEI at 109 and 229 days of gestation, respectively. The percentage of energy lost as feces varies mainly due to the quality of the diet. The energy loss in feces could reach 65% of GEI when the diet is mainly composed of low-quality forage. For diets containing large amount of processed grains, the proportion of GEI lost as feces can be less than 20% [25].

The daily methane production (liters) showed the same GEI pattern, with superior methane production for F1 HxG. However, when expressed as a percentage of GEI, no differences were observed between the genetic groups. Among the gestational ages, the percentage of GE lost as methane was greater (P<0.05) at 210 days for F1 HxG. In the Gyr group, the greater percentages of GE lost as methane were at 210 and 240 days of gestation. Although a concrete explanation for the fluctuation of methane production is limited by the available data, it is possible to suppose that the higher values for energy losses associated with methanogenesis are closely correlated with the digestibility of organic matter digestibility [7]. An equation proposed by [26] to estimate de methane production by zebu and crossbred animals in tropical conditions, demonstrate that the major factor influencing methane production is the amount of DMI and, no effects were of breed or physiological state were detected. Even with fluctuations between gestational ages, the methane production in relation to GEI is close to the 6.0% value proposed by [27] and, within the 5 to 12% range suggested by [28]. The daily losses of energy as urine did not differ between the genetic groups. The values of energy losses of urine in relation to GEI are above the minimal value of 3.0 to 5.0% proposed by [28]. Greater energy losses in urine can be justified by the greater excretion of nitrogenous compounds such as amino acids, creatinine and urea, which is typically 80 to 90% of the total amount of nitrogen in urine [29]. The carbon chain of amino acids can be used by the dam and foetus as glucose precursors after the deamination process. Several works [4, 30–32] indicate that gluconeogenesis is a metabolic pathway enhanced due to pregnancy and, thus, can increase nitrogen excretion during the third semester of gestation when the gravid uterus has a higher energy demand to support the accelerated rates of foetus development.

The deduction of the amount of energy lost in feces, urine and gases from the GEI results in the MEI. In the present study, the MEI (Mcal/d) for F1 HxG was greater (P<0.05) at all gestational ages. When expressed in kcal/kg BW^0.75^, MEI was 11% greater (P<0.05) for crossbred cows only after 240 days of gestation. Gyr cows showed no change in the total MEI over time. The mean value of obtained of 194.39 kcal/kg BW^0,75^ is closer to the value described by Ferrell et al. (1976a) for heifers submitted to a feeding level of 1.8x NE_m_. The ME for pregnancy (ME_p_) was obtained by subtracting the ME_m_ proposed by [15] from the MEI. No ME for maternal gain was assumed, in agreement with the restricted level of DMI defined. [4] calculated the ME_p_ using ME to NE_p_ conversion efficiency of 0.14 proposed by [3]. The authors estimated that the ME_p_ required by Holstein cows in the last month of pregnancy was 5.52 Mcal/d or 40 kcal/ kg BW^0,75^. In the present study, the ME_p_ was 5.33 and 4.46 Mcal/d for F1 H x G and Gyr cows, respectively. When expressed as kcal/kg BW^0.75^ the ME_p_ was 48.39 kcal/kg BW^0.75^ for crossbred cows and 46.61 kcal/kg BW^0,75^ for zebu cows. Both values are close to those values described by [4]. At 240 days the ME_p_ was greater for F1 HxG cows when compared to all other gestational ages of Gyr group (P<0.05).

The daily MEI proposed by [33] for Holstein cows in late gestation, with 550 kg BW (similar to the average BW of crossbred cows), was 17.80 Mcal/d. This is lower than the total MEI of F1 HxG cows. The same authors mention that for, Holstein cows with 450 kg of BW (similar to the BW of Gyr cows), the MEI to meet the requirements of maintenance and pregnancy was 15.3 Mcal/d. This value that is close to the total MEI of Gyr cows.

The percentage of total ME (ME_m_ + ME_p_) in relation to GEI corresponds to the metabolizability of the diet (q). This corresponded to 0.60 and was similar between genetic groups evaluated. [16] predicts a q_m_ (metabolizability for maintenance) of 0.53 for Friesian x Holstein cows fed diets with ME concentration of 2.40 Mcal/kg DM. The British Committee also suggests that, as the energy density of the diet increases, there is an increase in the values of “q” that can reach up to 0.64 for diets containing 2.90 Mcal ME/kg DM. The q_m_ behaves similarly for beef cattle, reaching values of 0.69 for diets containing 3.10 Mcal ME/kg DM [16]. [34] reported that the “q” of the diet depends on the quality of the feed and the energy balance to which the animal is subjected. That is, animals receiving high quality diet and subjected to low feeding level have a greater value of "q" compared to animals consuming low quality diet and subjected to high feeding level. The "q" of the diet consumed by the cows in this experiment supports this idea since they were subjected to a feeding level close to the maintenance requirements.

The relationship between ME and DE showed some significant variation between gestational ages, with no evident pattern. The values oscillated between 0.82 to 0.86. [16] suggests that this ratio is high, with a correlation between ME and DE equivalent to 82%. Indeed, the losses associated with methane and urine in this study were between 14 and 18%. The [35] adopts an ME/DE ratio of 0.80 and points to the fact that this value can vary considerably depending on the level of consumption, animal age and type of food. Confirming this, the [16] mentions that the ME/DE ratio can vary from 0.80 to 0.86 according to characteristics of the diet [6] used a fixed value of 0.82 to obtain the ME intake by pregnant F1 Holstein x Gyr cows, without considering any possible variations on this efficiency due to pregnancy or different gestational ages.

The HI may account for 20 to 30% of GEI for animals fed at maintenance level, 30% for lactating animals, and up to 66% for fattening animals [28]. The values obtained in the present agree with the ranges proposed by this author, for animals fed close to the maintenance level.

The heat produced in association with basal metabolism (NE_m_) should also be considered, as it inversely influences the energy retained by the animal thus altering productive performance. The NE_m_ (Mcal/d) for F1 HxG was greater than the NE_m_ for Gyr due to the greater BW of crossbred cows. When expressed as a proportion of GEI the NE_m_ did not differ between genetic groups or gestational ages (P = 0.06) as described by [15].

Another source of heat to be considered in pregnant animals is the HP from the gravid uterus. In general, the apparently inefficient use of ME for pregnancy results from the considerable energy cost for maintenance of products of conception, like the placenta. That is, oxidative metabolism is the major component of the total energy requirement of the conceptus. The disadvantage, in terms of energy efficiency, comes from oxidative reactions being exothermic, which means that much of the energy that could be directed to tissue development of the fetus is dissipated as heat. Also, the high fetal metabolic activity is responsible for a thermal gradient of 0.5 to 1.0°C between the mother and the fetus [36]. Apparently, there is relative hyperthyroidism (compared to the dam) described in ruminant fetuses that could explain their high metabolic rates [37].

Most of the parameters presented no interactions between genetic group and gestational age, with the exception for fed heat production and net energy for the development of the conceptus.

The energy efficiency of 13.0% to convert ME to net energy for pregnancy (NE_P_) is presented in Table 4; it did not vary among genetic groups or days of gestation. The low value of 14% proposed by the [5] and [3] is reported to be the energy efficiency use of ME by the gravid uterus (k_p_). The k_p_ value obtained in the present study is also close to values obtained by [6, 38, 39].

**Table 4:** Energy efficiencies utilization of F1 Holstein x Gyr (F1 HxG) and Gyr cows at 180, 210 and 240 days of gestation.

Item	Genetic group / Gestational age (d)						GG*GA^4^
	F1 HxG			Gyr			
	180	210	240	180	210	240	
q^1^	0.57	0.60	0.61	0.61	0.62	0.59	NS^5^
SEM	0.02	0.01	0.01	0.01	0.02	0.01	
ME/DE^2^	0.83^Bab^	0.82^b^	0.84^b^	0.86^Aa^	0.85^b^	0.84^b^	NS^5^
SEM	0.01	0.00	0.01	0.01	0.01	0.01	
k_p_^3^, %	13.93	12.92	10.71	16.79	13.98	9.72	<0.05
SEM^2^	2.67	1.78	5.00	3.27	3.58	4.18	

Means followed by different lowercase letters in the same row differ between times evaluated in the same genetic group. Means followed by different capital letters in the same row differ between genetic groups evaluated at the same gestational age.

^1^q = metabolizability (AFRC, 1993).

^2^ME/DE = metabolizable energy / digestible energy ratio.

^3^k_p_ = efficiency of metabolizable energy use for pregnancy (NE_p_ / ME).

^4^GG*GA = interaction between genetic group and gestational age.

^5^NS = non-significative (P>0.05).

There was no difference for the the efficiency of ME utilization data for pregnancy (k_p_) in our work should be applied mostly to the development of the conceptus (growth of fetuses and fetal membranes), since the heat coming from the uterus itself was already considered in the measurement of NE_m_ requirements using similar non-pregnant cows. According to [40] the composition of the uterus is relatively constant over gestation. Moreover, uterine fluids deposit is irrelevant in terms of nutrient uptake due to its exceptionally low DM content and constant composition during different gestational stages.

The NE_p_ did not differ among gestational ages and genetic groups and in this study include the energy associated gravid uterus components. The mean value of 2.52 Mcal/d is close to values estimated by equations of [5]. For 180, 210 and 240 days of gestation, NE_p_ calculated according to [5] equation is 2.47, 2.53 and 2.63 at 180, 210 and 240 days of gestation, respectively considering calf birth weight of 45 kg for Holstein cows. [6] using the comparative slaughter technique, found a NE_p_ (energy retained by the gravid uterus and mammary gland) for F1 HxG cows to be lower than those in the present study of 2.05, 2.12 and 2.19 Mcal/d at 180, 210 and 240 days of gestation, respectively, considering calf birth weight of 35 kg.

The knowledge of energy partition in zebu animals and their crosses is critical to better understand the digestive and metabolic processes that contribute to the great productive potential of these animals. Apparently, pregnancy does not influence dietary metabolizability (q) or significantly affect the relationship between ME /DE when cows consume diets close to maintenance level. As described for other breeds, the Gyr and F1 HxG also exhibited low energy efficiency processes involved in the conceptus development.

## Supporting information

S1 Data The raw data set used the conclusions drawn in a manuscript. (XLSX)
